# Papillomaviruses Use Recombination-Dependent Replication to Vegetatively Amplify Their Genomes in Differentiated Cells

**DOI:** 10.1371/journal.ppat.1003321

**Published:** 2013-07-04

**Authors:** Nozomi Sakakibara, Dan Chen, Alison A. McBride

**Affiliations:** Laboratory of Viral Diseases, NIAID, NIH, Bethesda, Maryland, United States of America

## Viral Life Cycle

**Figure 1:**
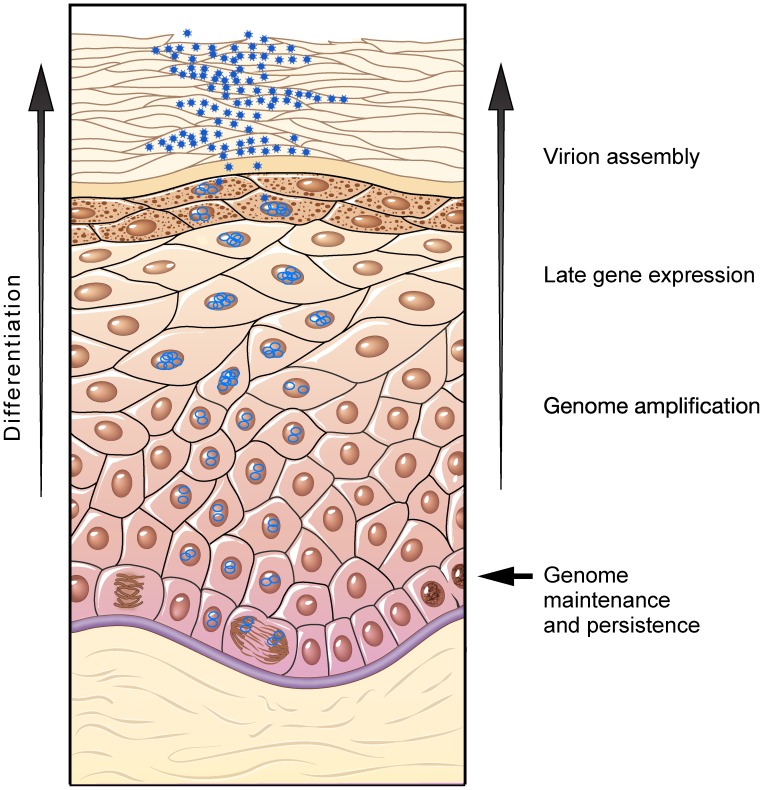
The papillomavirus life cycle is closely coupled with differentiation of the host epithelium. The virus infects the dividing basal cells through a microabrasion. The viral DNA is maintained at a low copy number in these cells. When basal cells divide, some daughter cells move up in the epithelium and begin the process of terminal differentiation. Papillomaviruses are finely tuned to this process and turn on late transcription, translation, and late DNA replication in specific stages of the differentiation process. Vegetative viral DNA replication takes place in cells that are in either the G2 phase of the cell cycle or have exited the cell cycle. By inducing the DNA damage response and homologous recombination repair pathways, the virus can efficiently replicate progeny genomes in differentiated cells without competition from host DNA synthesis.

Papillomaviruses have evolved a life cycle that is perfectly coordinated with the differentiation process of the host epidermal tissue. The stratified epithelium of the cutaneous and mucosal epithelium of the epidermis consists of a basal layer of cells (including stem cells) that continually divide to replenish the overlying layers of differentiated cells. Differentiation proceeds in a systematic process to generate the stratum spinosum, granulosum, and in some cases corneum to provide the epidermis with strength as well as a barrier against water loss and pathogen invasion (see Figure 1). Papillomaviruses capitalize on this process by infecting the cells of the stratum basale (through a microabrasion) and setting up a persistent infection in which the small double-stranded circular genome is maintained as a low copy replicating plasmid in the stratum basale. There is very little viral gene expression in these cells, just enough to replicate and maintain the viral genome, enhance cellular proliferation, and evade host immune defenses. However, when the infected cells begin the process of differentiation, late viral gene expression and viral genome amplification are induced. In this way, high-level viral transcription and replication is restricted to terminally differentiating cells (invisible to the immune system) and viral-laden squames are sloughed from the surface of the epithelium as part of the normal epidermal renewal process. One difficulty with this strategy is that the viruses need to synthesize large quantities of viral DNA in differentiated cells that have exited the cell cycle.

It has been thought that the E6 and E7 viral oncogenes papillomaviruses circumvented this problem by either inducing cell cycle reentry and/or preventing cells from leaving the cell cycle upon differentiation so that the virus could replicate its DNA in S-phase–like cells. However, a careful pulse-chase analysis of replication of cellular and viral DNA in differentiated tissue indicated that viral DNA was replicated after host DNA, most likely in the G2 phase of the cell cycle [1], [2]. Thus, the virus needs other means to obtain the machinery necessary to replicate viral genomes. There is accumulating evidence that papillomaviruses, like many other viruses, both induce and use the host DNA damage response for replication of viral DNA [3]–[7]. We propose that papillomaviruses use recombination-dependent replication (RDR) to produce progeny viral genomes in differentiated cells.

## Different Types of Replication

In the papillomavirus life cycle, it is generally assumed that there are at least three different phases of replication. The first occurs when the virion particle infects the basal keratinocyte. In this phase, the viral genome must undergo a few rounds of unlicensed replication to produce a small number of viral genomes in this initially infected cell. In the second phase, when the basal cells divide, the viral genomes replicate in concert with host DNA and are partitioned to daughter cells. Daughter cells can either remain in the basal layer and continue dividing, or can move upwards and begin the process of differentiation. Those cells that differentiate can activate late gene expression and the third phase of replication, vegetative viral DNA amplification. In this last phase of replication, large numbers of progeny viral genomes are synthesized for packaging in viral capsids. Thus, the infected basal cells provide a long-term reservoir of infected cells that are continually replenishing the overlying epithelium and producing viral particles (reviewed in [8]).

Papillomaviruses encode two proteins that are directly involved in viral DNA replication, and the well-defined origin of replication contains adjacent binding sites for these proteins [9], [10]. The E1 protein is an ATP-dependent helicase that binds specifically and cooperatively to the replication origin with the E2 protein. E2 is a transcriptional regulator that also functions to load the E1 helicase on the origin during initiation of replication [11]. After loading E1 on the origin, E2 is displaced from the complex and E1 converts to a hexameric helicase that encircles and unwinds the viral DNA to allow access of cellular replicative proteins. In the maintenance phase of replication, E2 tethers the viral genomes to host chromosomes to maintain them in dividing cells and partition them to daughter cells (reviewed in [12]).

Both E1 and E2 are required for initial and vegetative amplification of viral DNA [13]. In fact, HPVs induce caspases in differentiated cells that cleave the N-terminus of the E1 protein to promote vegetative viral DNA replication [14]. It has been assumed that both E1 and E2 proteins are required during all three phases of replication and that E2 partitions the viral genomes during the maintenance phase of replication. However, there is some evidence that E1 might not always be required during the maintenance phase [13], [15]. This is not surprising, as E1 is very toxic to cells and must be sequestered in the cytoplasm except when required for replication [5], [16], [17]. To date, all evidence indicates that the E2 protein is essential for genome maintenance [18]. However, one could envisage circumstances in which the viral genome could be replicated and partitioned by host proteins in infrequently dividing cells. For example, although not yet well established for HPV, there are emerging studies that indicate that papillomaviruses might develop true latent infections in which the viral genome is retained in a silent state with little or no gene expression [19]–[21].

## Papillomavirus Replication and the DNA Damage Response

DNA replication of many viruses induces a DNA damage response because viral DNA and replication intermediates are sensed by the host as damaged DNA [22], [23]. Viral proteins can also induce a DNA damage response by interfering with cell cycle regulation and inducing replication stress [24]. Viruses are equipped to deal with such host responses and can intercept or inactivate any signal that might be detrimental to viral DNA replication. But viruses also rarely miss an opportunity to hijack and take advantage of host defenses, and many use the ATM (ataxia telangiectasia mutated) and ATR (ATM and Rad3-related) arms of the DDR responses for various aspects of viral replication (reviewed in [25]).

Human papillomaviruses (HPVs) induce an ATM-mediated DNA damage response in the differentiated cells of infected epithelial tissue, and this response is required for efficient vegetative viral DNA replication [3]. Moody and Laimins provided the first indication that papillomaviruses used the cellular DNA damage response and, importantly, these studies were carried out in naturally infected HPV31-infected cells derived from a cervical lesion [3]. These cells maintain the viral genome as an episome and upon differentiation support the complete viral life cycle [26], [27].

Subsequent studies have examined the role of individual viral proteins in facilitating viral replication by inducing the DNA damage response in both keratinocytes and other cell types. The E7 protein plays a role in activation of the ATM response both directly [3] and indirectly by causing replication stress due to a deficiency in nucleotides [24]. The E1 protein also induces a DNA damage response that requires both the DNA binding and helicase functions of the protein [5]–[7], [28]. When expressed together, both E1 and E2 are localized to prominent nuclear foci that recruit markers of the DNA damage response and repair pathways [5]–[7], [29]. In the presence of E1, the E2 protein nucleates these foci onto host chromatin [6]. In this situation, the E1 protein is responsible for inducing the DNA damage response, perhaps by locally melting regions of host DNA [7]. The E1-E2 foci appear very similar to those that accumulate in differentiated tissue, in that they recruit DNA damage response markers such as pATM, pChk2, γH2AX, MRE11, and NBS1. In the presence or absence of viral DNA, the E1-E2 foci incorporate labeled nucleotides, even though the cells are not in S-phase, indicating that active DNA synthesis or repair is taking place [6], [29].

Thus, both E7 and E1 proteins can induce the DNA damage response pathways. The usual outcome of DDR activation is cell cycle arrest, which would not be beneficial for many viruses. However, since papillomaviruses seem to only highly activate this response in differentiated keratinocytes, cell cycle arrest has no negative consequences for the virus. Resting or arrested cells are well equipped with specific enzymes (e.g., p53R2 ribonucleotide reductase) to obtain deoxyribonucleotides for DNA repair [30].

## Recombination-Dependent Replication

When the DNA damage response is activated in response to cellular DNA damage or the collapse of a replication fork, the cell must decide whether the damage can be repaired and select the means of repair. Mammalian cells repair DNA breaks either by nonhomologous end joining (NHEJ) or by homologous recombination (HR). In NHEJ, the broken ends are aligned and ligated (often resulting in mutation). This can take place at any stage of the cell cycle since a complementary sequence template is not required. Conversely, HR requires a homologous sequence (most often the sister chromatid) and so occurs only in the post-replicative phases of the cell cycle (late S and G2). In homologous recombination, the broken end of DNA is resected to produce a single-stranded 3′ end that invades the double-strand of the homologous template to initiate DNA synthesis. The advantage of this type of repair is that it is highly efficient and faithful as it uses replicative DNA polymerases to duplicate a homologous DNA strand [31], [32]. During the homologous recombination process, long stretches of DNA are resected and synthesized and ample substrates and proteins required for DNA synthesis must be recruited to the site of damage. The DNA damage response signaling cascade promotes an influx of the necessary components to DNA damage foci. Clearly, it would be very advantageous for a virus to mimic and hijack this process. By initiating the DDR response in the vicinity of the viral genome, all components required for DNA synthesis would be delivered to the genome and the DNA damage response foci would become viral replication factories. Additional advantages are that this can occur in the G2 phase of the cell cycle (in differentiated cells) where there is no competition from host DNA synthesis.

## Evidence for Homologous Recombination in Papillomavirus Replication

Papillomaviruses replicate in a bidirectional theta mode, at least in the maintenance phase of the life cycle. However, there have been several observations of additional replication intermediates that are consistent with rolling circle replication [33]–[35]. Flores and Lambert have shown that there is a fundamental shift in the mode of replication of both HPV16 and HPV31 from bidirectional theta replication in undifferentiated cells to an alternative mode in differentiated cells. 2D gel analysis has demonstrated that in differentiated cells replication is unidirectional and a single initiation event can give rise to multiple copies of the viral genome [33]. Although characteristic of rolling circle replication, these intermediates are also consistent with recombination-dependent replication. Lariat-containing genomes have also been isolated from a small percentage of BPV1 virion particles, which is consistent with a different mode of replication in the late stages of infection [34]. And, although viral DNA isolated from HPV11-containing respiratory papillomas showed predominantly theta mode replication intermediates, a small number of molecules were observed that were consistent with other mechanisms such as rolling circle [36].

**Figure 2:**
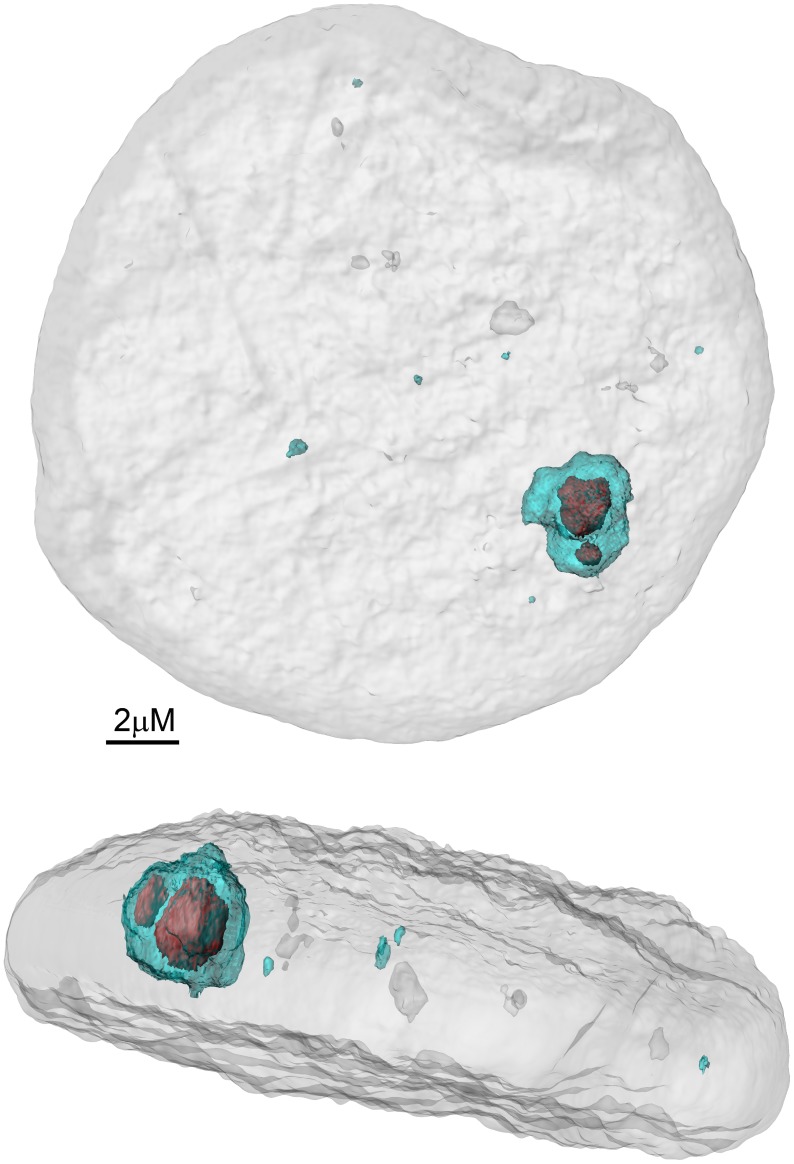
CIN612-9E cells were derived from a cervical lesion and contain hundreds of copies of extrachromosomally replicating HPV31 genomes [**52**] **.** These cells can be induced to differentiate with high calcium–containing medium, which switches on vegetative viral DNA replication [3]. Many of these cells contain multiple small replication foci [3], [4]; but numerous cells contain one large foci, as shown here, perhaps indicative of a temporal evolution. The nucleus shown has been stained with DAPI (grey) and antibodies to γH2AX to identify the viral replication foci (shown in cyan), and RAD51 to identify centers of homologous recombination (shown in red). 3D reconstruction of Z-stacks of confocal images was performed using Bitplane Imaris.

Analysis of the composition of the large replication foci generated by calcium-induced differentiation of HPV genome–containing cells shows that they contain proteins consistent not only with the DNA damage response, but also with factors involved in homologous recombination. One of the key players in the recombination process is Rad51, a recombinase that coats the single-stranded resected DNA and promotes its invasion into the homologous template (reviewed in [37]). As shown previously by Gillespie and Moody, and here in Figure 2, Rad51 colocalizes with HPV replication foci. γH2AX is incorporated into the chromatin surrounding the site of damage in cellular DNA damage foci, and colocalizes with viral DNA in HPV replication foci [4]. As shown in Figure 2, Rad51 forms punctate foci within a cloud of γH2AX, indicating that these are most likely the core sites of initiation of viral DNA synthesis. The HPV-differentiated cell replication foci also stain with Rad52 and pNBS1, which are also all involved in various aspects of homologous recombination [4].

## Many Viruses Use Recombination-Dependent Replication as One of Their Modes of Replication

Many viruses use RDR as one of their modes of replication and often encode many of the gene products required for this mode of replication [38]. Common factors often encoded by dsDNA viruses are a recombinase, a recombinase mediator and/or single-stranded DNA binding protein, an exonuclease for end resection, a Holliday junction resolvase, and a replicative DNA helicase [38]. Best characterized is T4 bacteriophage replication, where gene products required for each step of RDR have been well defined (reviewed in [38]–[40]). T4 encodes proteins with functions similar to those involved in homologous recombination of higher eukaryotes. For example, T4 encodes a protein analogous to the Rad51 recombinase, the RPA single-stranded DNA binding protein, and a recombination mediator protein that functions similar to Rad52, Rad51 paralogs, and BRCA2 [40]. The mammalian viruses HSV1 and vaccinia also use RDR in part of their life cycle and encode several proteins involved in RDR replication (reviewed in [38], [41]). Large viruses such as bacteriophages, baculoviruses, vaccinia virus, and HSV encode most proteins required for viral DNA replication, giving them independence from the host cell. Conversely, papillomaviruses are very dependent on host factors and so it is very likely that, for the most part, papillomaviruses use and modify host recombination factors for replication.

## Mechanism of Papillomavirus Recombination-Dependent Replication

Papillomaviruses are evolving very slowly, with an evolutionary rate of approximately 2×10^−8^ nucleotide substitutions per site per year for the coding region of the virus [42]. Thus, the host polymerases that duplicate the viral DNA must be of high fidelity. Homologous recombination results in virtually error-free DNA synthesis as it often employs the high fidelity replicative DNA polymerases, delta and epsilon [31]. HR mechanisms can be divided into several modes, including double Holliday junction (dHJ), synthesis-dependent strand annealing (SDSA), and break-induced recombination (BIR). In BIR, the end of the invading single-strand (often derived from a collapsed replication fork) can be extended for hundreds of kilobases using replicative factors [32], [43], making this a potentially highly efficient mode for vegetative viral DNA replication. BIR is also unidirectional (as observed for the alternative mode of HPV16 replication [33]) and would give rise to long concatamers of viral DNA that could be processed to unit-sized genomes. Also, as noted previously [6], BIR pathways do not require origin licensing proteins to reinitiate DNA replication [32] and so can be very efficient for a virus.

The exact mechanism by which papillomaviruses manipulate and use the homologous recombination pathways remains to be elucidated. One possibility is that it follows a model similar to that proposed for the circular bacteriophage SPP1 [38]. In this model, replication switches from theta mode to RDR when replication collapse or blockage gives rise to a DNA end that is promoted to invade another supercoiled molecule by the HR machinery. The resulting D-loop forms a bubble that migrates in a unidirectional fashion around the viral genome, generating long concatamers by coupling recombination and replicative activities. The role of the E1 and E2 proteins in this process is not clear as there is less need for origin-specific replication initiation proteins in such a model. However, RDR often employs replicative helicases, such as phage T4 Gp41, and its helicase loader, Gp59, to coordinate recombination and replication and leading- and lagging-strand DNA synthesis (reviewed in [37]). Elucidation of novel functions of the papillomaviral E1 and E2 proteins could lead to the development of novel therapies for HPV infection.

Viruses that that replicate via long concatameric intermediates must resolve these intermediates into unit-sized viral genomes. Large viruses such as vaccinia or herpes simplex encode factors that assist in this process [44], [45]. However, the strategy of small viruses is to manipulate or adapt host factors for viral use. Therefore, an alternative, or additional, role for cellular homologous recombination is to recombine and resolve papillomavirus DNA into circular, unit-sized genomes.

## Clinical Association of HPV Replication and DNA Repair Pathways

One clinical connection between HPV oncogenesis and DNA repair is Fanconi anaemia (reviewed in [46]). Fanconi anaemia (FA) results from the mutation in any one of 15 genes in the FA repair pathway and gives rise to increased susceptibility to cancers and hypersensitivity to agents that cause inter-strand DNA crosslinks. Notably, individuals with FA are extremely susceptible to head and neck squamous-cell carcinomas, which have about a 25% association with HPVs in normal individuals. There are reports of a greatly increased association of HPVs with these carcinomas in FA, but this is controversial (reviewed in [47]). Nonetheless, HPV16 E7 greatly increases keratinocyte proliferation in cells deficient in FANCD2 or FANCA, and there is a substantial increase in levels of viral DNA in differentiating cells [48], [49]. This seems counterintuitive to the model proposed here, but there is recent evidence that the role of the FA pathway is to direct repair to the more faithful HR pathway instead of the more error-prone NHEJ pathway [50], [51]. Thus, it is possible that viral DNA synthesized in cells defective in the FA repair pathway is prolific, but aberrant, and perhaps more likely to become integrated into the host genome.

## Advantages of RDR Replication for Papillomaviruses

Papillomaviruses have evolved a remarkable lifestyle in which they take advantage of many fundamental processes involved in the growth and differentiation of stratified epithelia. They initially bind to a wounded epithelium, interacting with proteoglycans exposed on the basement membrane before entering the adjacent keratinocytes that have been stimulated to divide by the injury [8]. The virus establishes a long-term home within these cells by closely associating its genome with host chromatin while greatly limiting viral gene expression to escape detection by the host. The virus waits until its host cells have begun to differentiate and have safely escaped immune surveillance before it switches to late replication and gene expression. Although the differentiated host cells have completed S-phase, the virus induces or mimics a DNA damage response in the vicinity of the genome, resulting in a cascade of recombination and replicative factors being delivered to a nascent viral replication factory. The DDR is a very potent signaling mechanism that can detect a single nucleotide mutation or collapsed fork and immediately build a repair foci consisting of a multitude of signaling and repair factors. The nucleation process is extremely efficient and allows the virus to replicate without competition from host DNA syntheses. Hijacking the host DNA damage response and HR repair processes is just one more instance of the ingenious strategies acquired by this ancient group of persistent viruses.
